# An intron-derived motif strongly increases gene expression from transcribed sequences through a splicing independent mechanism in *Arabidopsis thaliana*

**DOI:** 10.1038/s41598-019-50389-5

**Published:** 2019-09-24

**Authors:** Jenna E. Gallegos, Alan B. Rose

**Affiliations:** 1 Department of Molecular and Cellular Biology, University of California, Davis, CA 95616-8535 USA; 2 Present Address: Department of Chemical and Biological Engineering, Colorado State University, Fort Collins, CO 80523-1370 USA

**Keywords:** Molecular engineering in plants, Transcriptional regulatory elements, Plant molecular biology

## Abstract

Certain introns significantly increase mRNA accumulation by a poorly understood mechanism. These introns have no effect when located upstream, or more than ~1 Kb downstream, of the start of transcription. We tested the ability of a formerly non-stimulating intron containing 11 copies of the sequence TTNGATYTG, which is over-represented in promoter-proximal introns in *Arabidopsis thaliana*, to affect expression from various positions. The activity profile of this intron at different locations was similar to that of a natural intron from the *UBQ10* gene, suggesting that the motif increases mRNA accumulation by the same mechanism. A series of introns with different numbers of this motif revealed that the effect on expression is linearly dependent on motif copy number up to at least 20, with each copy adding another 1.5-fold increase in mRNA accumulation. Furthermore, 6 copies of the motif stimulated mRNA accumulation to a similar degree from within an intron or when introduced into the 5′-UTR and coding sequences of an intronless construct, demonstrating that splicing is not required for this sequence to boost expression. The ability of this motif to substantially elevate expression from several hundred nucleotides downstream of the transcription start site reveals a novel type of eukaryotic gene regulation.

## Introduction

Significant efforts have been invested in identifying the DNA sequences that control the expression of individual genes in eukaryotes. These studies have revealed many common kinds of regulatory elements that collectively constitute promoters in the broadest sense of the term. These include the sites surrounding and immediately upstream of the transcription start sites (TSSs) to which general transcription factors bind to form the pre-initiation complex, proximal binding sites (usually within 1 kb upstream of the TSS) for regulatory transcription factors, and distal elements, such as enhancers, which can affect expression over great distances in either direction (reviewed in^1–5^).

In addition to these well-known regulators of transcription, other transcribed sequences can play an important role in controlling expression. 5′ and 3′ UTR’s have been shown to influence mRNA stability, export, and translation (reviewed in^6–9^), exons can contain transcription factor binding sites^10^ or intragenic enhancers^11^, and introns have been shown to affect gene expression by a number of known and unknown mechanisms collectively known as intron-mediated enhancement (IME)^12^.

Some introns contain enhancers^13,14^, alternative transcription start sites^15^, or transcription factor binding sites^16^. In addition, splicing can have a general positive effect on expression via coupling with other mRNA processing events such as capping and polyadenylation^17^. Deposition of the exon junction complex proteins also aids in mRNA export and translation^18,19^ and splicing can influence transcription by affecting the phosphorylation state of RNA polymerase II^20^.

In one specific type of IME, certain introns increase mRNA accumulation by a poorly understood mechanism^21^. These mRNA-increasing introns must influence expression in a manner that is mechanistically distinct from enhancers or proximal promoter elements because the ability of natural introns to affect expression when moved to new locations is unlike that of either promoters or enhancers. The first intron from either the Arabidopsis *UBQ10* or *TRP1* gene stimulates mRNA accumulation from 218 nt downstream of the start codon of a *TRP1:GUS* reporter construct, and to a slightly lesser extent from 292 nt further downstream, but neither intron affects mRNA accumulation when 1095 nt or more from the ATG^22^. In addition, the *UBQ10* first intron (hereafter ‘the *UBQ10* intron’) increases expression from either of two locations in the 5′-UTR (at −18 or −84) but has no effect when upstream of the TSS^22,23^. The observation that many introns stimulate gene expression only from within transcribed sequences near the 5′-end of a gene^22,24–26^ is the basis for the IMEter algorithm described below.

The role of intron splicing in this unique mechanism of IME remains the subject of some debate^21^. Splicing is clearly not sufficient to increase mRNA accumulation, because many efficiently spliced introns have no effect on expression^27,28^. Testing whether or not splicing is necessary is complicated by the fact that disrupting splicing has many consequences. Constructs with an unspliceable intron produce mRNA that differs in size and structure from constructs containing a spliceable intron or an intronless control, and therefore may differ in stability. The intron sequences retained in the mRNA can also cause frame shifts or contain premature start or stop codons, all of which might abolish translation of the reporter gene and lead to mRNA instability through nonsense-mediated mRNA decay. In cases where splicing was prevented but the reading frame was preserved by adjusting intron length and eliminating in-frame start and stop codons, expression levels were reduced but not eliminated^27,29–31^. The degree to which expression levels dropped varied greatly by species and the size, location, and original stimulating ability of the intron, precluding broad conclusions about the need for splicing in IME.

The differing ability of spliced introns to increase mRNA accumulation implies that some must contain stimulating sequences that others lack. These sequences have proven difficult to identify because they are redundant and dispersed throughout stimulating introns^29,32^. Progress was made using the IMEter algorithm, which generates a score that reflects the degree to which the oligomer composition of a given intron resembles that of promoter-proximal introns genome-wide^32^. High IMEter scores have accurately predicted the stimulating ability of introns in Arabidopsis^32^, soybeans^33^, and other angiosperms^34^. The IMEter does not directly reveal stimulating sequences but can be used to identify sufficient numbers of potentially stimulating introns to allow computational searches for shared sequences.

One such motif, TTNGATYTG, was found to be over-represented in introns with high IMEter scores in Arabidopsis^32^. Rearranging nucleotides to create 6 or 11 copies of this motif converted a non-stimulating intron from the Arabidopsis *COR15a* gene into one that boosts mRNA accumulation 13- or 21-fold, respectively^35^. Introns containing this motif behave similarly to the *UBQ10* intron in that they increase mRNA accumulation even in the absence of the proximal promoter and can influence the location of the TSS^23^. Together these data suggest that the sequence TTNGATYTG is sufficient to create an intron that boosts mRNA accumulation by the same mechanism as the *UBQ10* intron.

To further test the similarity in mechanism by which the TTNGATYTG motif and the *UBQ10* intron affect expression, the ability to increase mRNA accumulation of the *COR15a* intron containing 11 copies of the motif was determined at various locations within a *TRP1:GUS* reporter gene. Additional parameters explored were whether or not the motif increased expression in a dose-dependent manner, if there was an upper limit in the degree to which mRNA accumulation could be stimulated, and whether or not the mechanism requires splicing, and therefore must be specific to introns. Here we report that the positional requirements of the increase in expression caused by the TTNGATYTG motif were very similar to those of the *UBQ10* intron, that the effect of the motif on expression was surprisingly linear up to at least 20 copies of the motif, and that the motif stimulated mRNA accumulation to a similar degree from within exon sequences of an intronless construct or in an intron, demonstrating that splicing is not required.

## Results

### The TTNGATYTG motif stimulated expression over a limited range

A defining characteristic of natural introns that boost mRNA accumulation is that they only affect expression when located within the first Kb or so of transcribed sequences^22^. To determine if the positional requirements of the TTNGATYTG motif were similar to those of the *UBQ10* intron, the ability of an intron engineered to contain 11 copies of the motif to stimulate expression was tested from select locations within a *TRP1:GUS* reporter construct. This intron, which was previously generated and designated *COR15a*11L^35^, was created by rearranging sequences within a naturally non-stimulating intron from the *COR15a* gene^27,32^. The *COR15a*11L intron was inserted into *TRPI:GUS* fusion constructs at one of five positions: upstream of the transcription start site (−321 relative to the A of the ATG translational start codon), within the 5′ UTR (−18), at the 3′ end of sequences derived from *TRP1* exon 1 (+218), near the middle of the *GUS* gene (+1095), or towards the 3′ end of the *GUS* gene (+1834, Fig. 1a). *TRPI:GUS* constructs containing the *COR15a*11L intron at the different locations were transformed into Arabidopsis and expression levels were compared in homozygous single-copy transgenic lines (see Supplementary Table S1). The data shown for the *UBQ10* intron are only from lines tested at the same time as the *COR15a*11L intron and are in good agreement with previously published results^22,23,35^ (see Supplementary Table S2).

**Figure 1:**
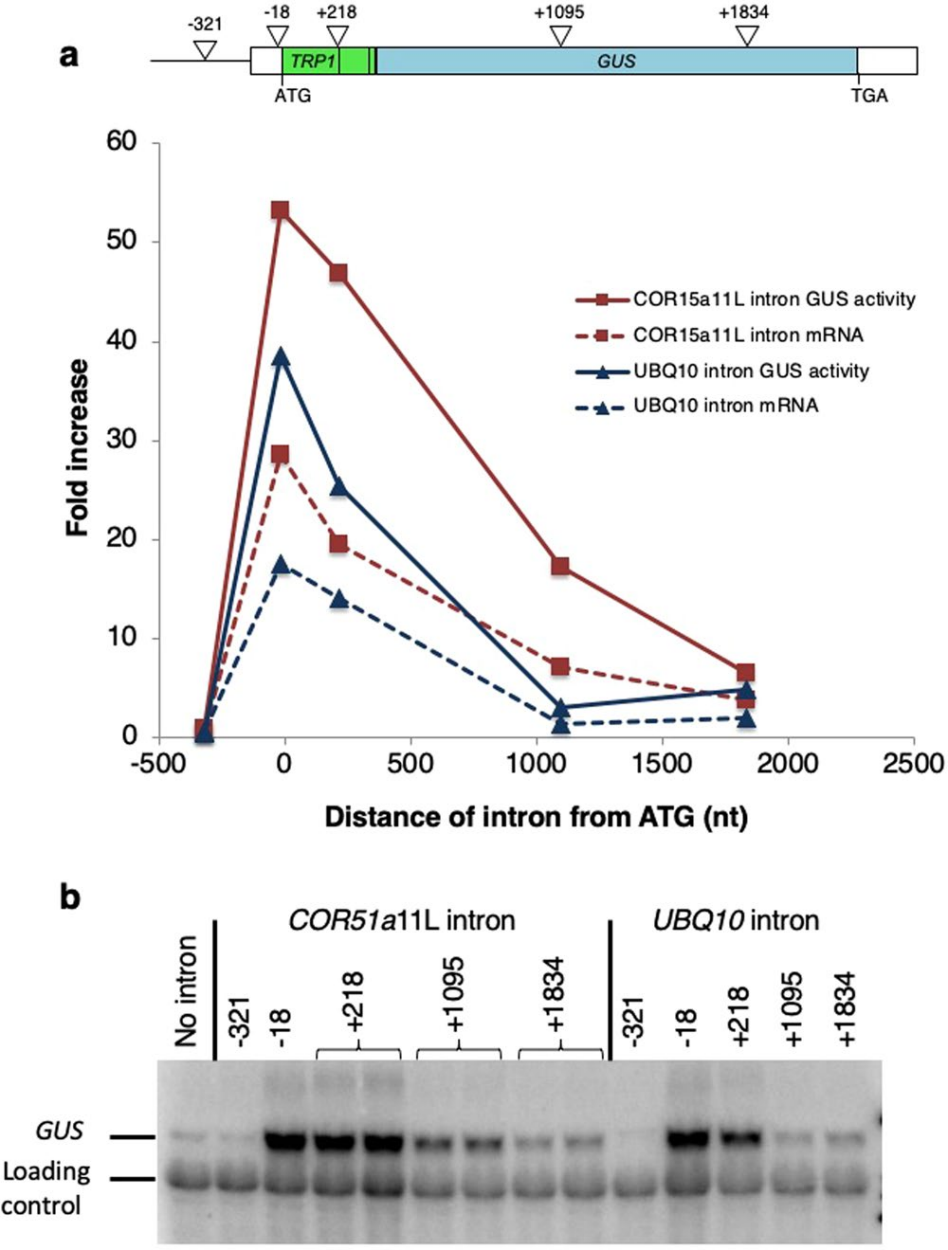
Comparing the effect on expression of the *UBQ10* and *COR15a*11L introns at different locations. (**a**) Top. Map of the *TRPI:GUS* fusion with the sites of intron insertion indicated by triangles. Numbering is relative to the A of the *TRP1* start codon. Bottom. Average GUS enzyme activity and mRNA accumulation in single-copy lines containing either the *UBQ10* or the *COR15a*11L intron at the specified locations. See Supplementary Table S1 for detailed expression data. (**b**) Representative RNA gel blot probed with *GUS* and a loading control (the endogenous *TRP1* gene). The uncropped blot image is shown in Supplementary Fig. S1.

Overall, the activity profile of the *COR15a*11L intron was very similar to that of the *UBQ10* intron (Fig. 1a). Neither intron increased either GUS activity or mRNA accumulation when located upstream of the TSS. Both introns had the largest effect on expression from within the 5′-UTR at −18 relative to the ATG. The activity of both introns declined at a similar rate as they were moved downstream. The main differences between them are that the *COR15a*11L intron had approximately twice the effect on expression as the *UBQ10* intron at comparable locations near the start of the gene, and the *COR15a*11L intron still had a substantial effect on expression from the +1095 position but the *UBQ10* intron did not (Fig. 1). The lack of stimulating effect of the *COR15a*11L intron from the 3′ end of the *TRP1:GUS* fusion or upstream of the TSS rules out the possibility that the TTNGATYTG motif acts as a conventional enhancer, and strongly suggests that it increases mRNA accumulation by the same mechanism as the *UBQ10* intron.

### The TTNGATYTG motif stimulated expression in a dose-dependent manner

The ability of the *COR15a*6L and *COR15a*11L introns to increase mRNA accumulation is proportional to the number of TTNGATYTG motifs they contain^35^. To generate a more complete picture of the stimulating effect, four additional derivatives of the *COR15a* intron were tested that contain 3, 8, 15 or 20 copies of the TTNGATYTG motif. The *COR15a*3L and *COR15a*8L introns contain all but three of the motifs present in the *COR15a*6L and *COR15a*11L introns, respectively. Because the motifs are made by rearranging nucleotides, the *COR15a*, *COR15a*3L, 6 L, 8 L, and 11 L introns are each 306 nt long and are composed of 93 A, 35 C, 44 G, and 134 T nucleotides. There are only 11 regions within the *COR15a* intron that can be shuffled to match the sequence TTNGATYTG. To make the *COR15a*15L and *COR15a*20L introns, either 4 or 9 copies of the sequence TTAGATCTG were inserted into the *COR15a*11L intron, resulting in somewhat longer introns (342 nt and 387 nt). Each intron was placed at the 3′ end of sequences derived from *TRP1* exon 1 (+218) of a *TRP1:GUS* fusion, which were introduced into Arabidopsis by *Agrobacterium*-mediated transformation.

**Figure 2:**
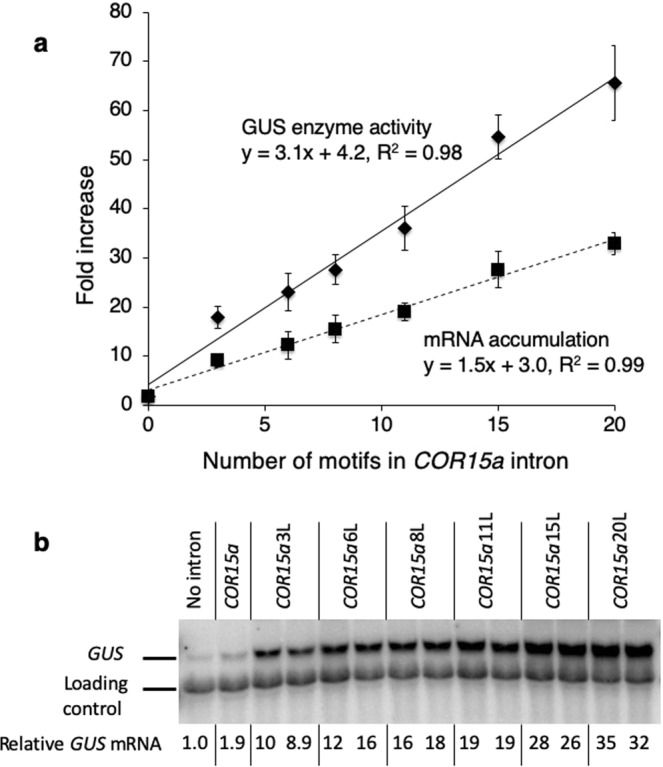
Comparing the effect on expression of the *COR15a* intron modified to contain different numbers of the TTNGATYTG motif. (**a**) Average GUS enzyme activity and mRNA accumulation in single-copy lines containing a *TRP1:*GUS fusion with a *COR15a* intron modified to contain the indicated number of TTNGATYTG motifs at the 3′ end of *TRP1* exon 1 sequences (+218 in Fig. 1). See Supplementary Table S2 for detailed expression data. (**b**) Representative RNA gel blot probed with *GUS* and a loading control (the endogenous *TRP1* gene). Each adjacent lane with the same label represents an independent single-copy homozygous line. The uncropped blot image is shown in Supplementary Fig. S2.

As shown in Fig. 2 and Supplementary Table S3, the GUS enzyme activity and mRNA accumulation in single-copy lines was proportional to number of TTNGATYTG motifs in the *COR15a*-derived intron in the *TRP1:GUS* fusion. The *COR15a*20L intron increased steady state *GUS* mRNA levels more than 30-fold, and GUS enzyme activity nearly 70-fold. The straightness of the line suggests that the effects of the motifs were strictly additive, and the slope indicates that each copy of the motif contributed an additional 1.5-fold increase in mRNA accumulation. An upper limit to the degree to which copies of this motif could potentially increase mRNA accumulation could not be estimated because the effect of the motifs remained linear throughout the range tested.

### The TTNGATYTG motif stimulated expression from within exon sequences of an intronless gene

**Figure 3:**
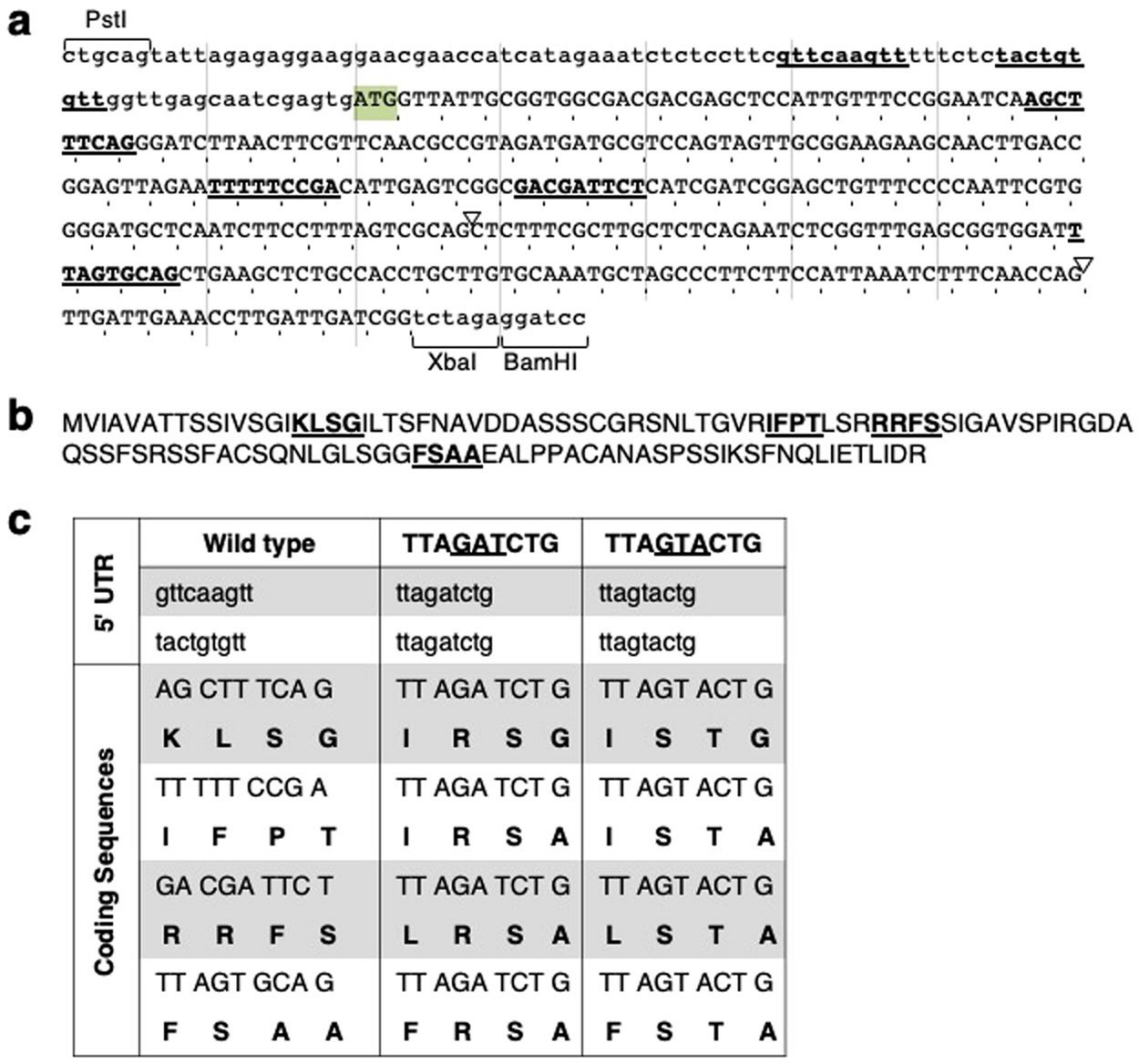
Details of changes to *TRPI:GUS* sequence to introduce motifs. (**a**) Uppercase letters mark *TRPI* coding sequences with the start codon highlighted. Lowercase letters indicate the *TRPI* 5′ UTR and the sites used to fuse *TRP1* to *GUS*. The inverted triangles show the location of introns in the endogenous *TRP1* gene. Sequences that are underlined and bold were changed to either TTAGATCTG or TTAGTACTG. (**b**) The amino acids encoded by the *TRPI* sequence shown in A, with the regions affected by introducing the motifs underlined and in bold. (**c**) Details of the nucleotides and amino acids changed.

To determine if sequences involved in IME can stimulate expression in the absence of splicing, six copies of the sequence TTAGATCTG (the most active tested version of the TTNGATYTG motif^35^) were engineered into the first 450 nt of transcribed sequences of an intronless *TRP1:GUS* fusion (Fig. 3). The *TRP1* sequences are described as exons 1, 2, or 3 based on their location in the endogenous *TRP1* gene. Five motifs were introduced into *TRP1* exon 1 sequences, two of which were in the 5′ UTR, and one copy was introduced into *TRP1* exon 2 sequences (Fig. 3). As a negative control, a second *TRP1:GUS* fusion was generated in which the AT dinucleotide at the center of each motif was changed to TA, making the motif TTAGTACTG. This small inversion was previously shown to eliminate virtually all of the motif’s effect on mRNA accumulation from within an intron^35^.

**Figure 4:**
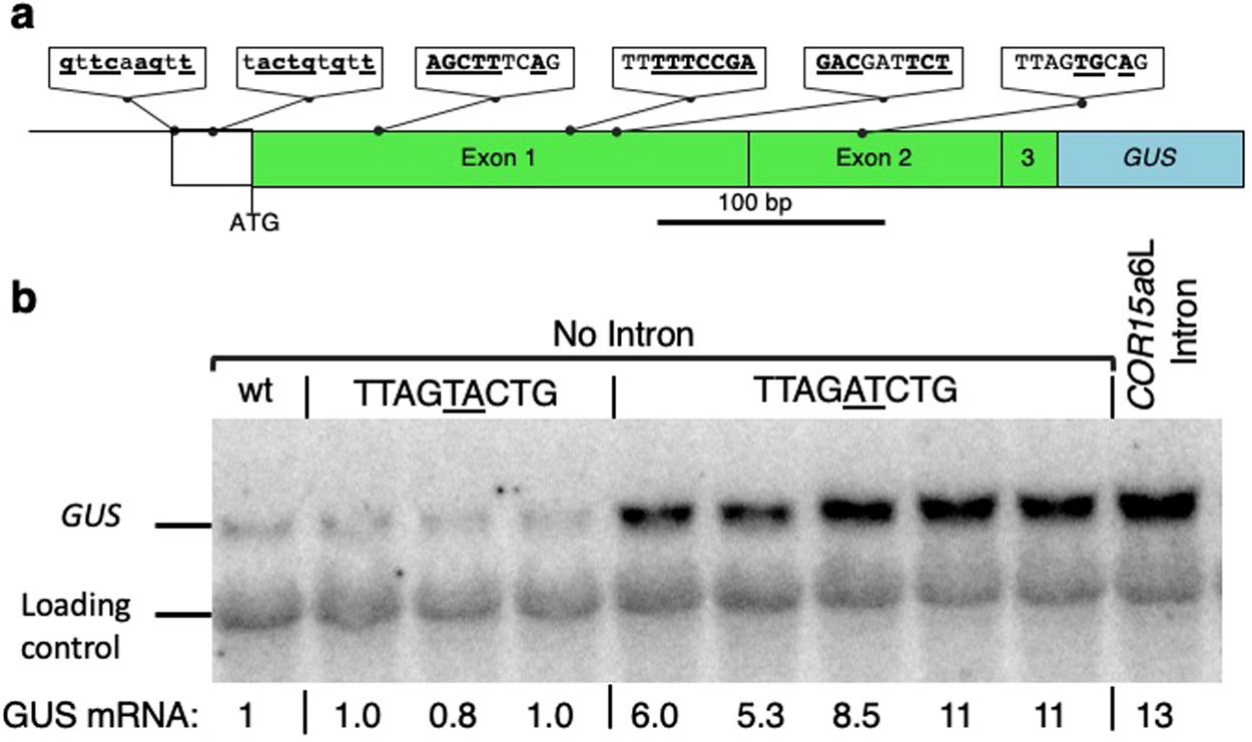
Testing the ability of the TTNGATYTG motif to stimulate expression from within exons. (**a**) The bold and underlined nucleotides in the sequences at the indicated locations in a *TRP1:*GUS fusion were changed to match the motif. The designations of exons 1, 2, and 3 refer to the location of the same sequences in the endogenous *TRP1* gene. The *COR15a*6L intron in the control construct is located at the 3′ end of *TRP1* exon 1 sequences. No other constructs contain an intron. (**b**) RNA gel blot probed with *GUS* and a loading control (the endogenous *TRP1* gene). Each adjacent lane with the same label represents an independent single-copy homozygous line. See Supplementary Table S4 for detailed expression data. The uncropped blot image is shown in Supplementary Fig. S3.

Locations for introducing the motifs were selected to minimize changes to mRNA and protein structure (Fig. 3). Existing sequences were searched for nine contiguous nucleotides composed of two As, one C, two Gs, and four Ts. Sequences that matched this criterion, or differed by no more than two nucleotides, were rearranged into the sequence TTAGATCTG or TTAGTACTG. In this way, the mRNAs from the tested constructs and controls would remain virtually unchanged in GC content and length. The first two exons of the *TRP1* gene encode a chloroplast transit peptide^36^, which are poorly conserved and are cleaved off during chloroplast import^37^. Motif insert locations were selected to maximize the degree to which the changes in amino acids were conservative and consistent with the composition of chloroplast transit peptides. Therefore, the changes made to introduce motifs were expected to have minimal effects on the activity of the mature GUS protein. The stimulating ability of the motif in exonic locations was compared with that of the *COR15a6L* intron located between *TRP1* exon 1 and exon 2 sequences. Expression was measured in single-copy transgenic Arabidopsis at both the mRNA and enzyme activity level and compared to intronless *TRP1*:*GUS* controls (Fig. 4, Supplementary Table S4).

Single copy lines containing the intronless *TRP1:GUS* fusion with six copies of the TTAGATCTG motif in exons accumulated on average 7.3 times more *TRP1:GUS* mRNA than did the unmodified intronless control (Fig. 4, Supplementary Table S4). This is slightly less than the 9.6 fold increase in expression caused by six copies of the same motif within the *COR15a*6L intron. In contrast, the fusion containing the TTAGTACTG motif produced about the same amount of mRNA as the unmodified intronless control. Therefore, the increase in mRNA accumulation caused by the TTAGATCTG motif is similar in intronic and exonic locations, indicating that this motif did not need to be located within an intron to boost expression.

## Discussion

The TTNGATYTG motif, which was identified computationally as being overrepresented in introns with high IMEter scores, was previously shown to be capable of converting the formerly non-stimulating *COR15a* intron into one that strongly increases mRNA accumulation^35^. Here we showed that the expression-stimulating properties of introns that contain this motif are very similar to those of naturally occurring introns but are unlike enhancer elements. The *UBQ10* and *COR15a11L* introns both stimulated mRNA accumulation only from within transcribed sequences near the 5′ end of a gene. This locational specificity, together with the observation that the *UBQ10*, *COR15a*6L and 11L introns all activate expression of a construct in which the normal transcription start sites were deleted^23^, strongly suggests that the TTNGATYTG motif is sufficient to increase mRNA accumulation by the same mechanism as the *UBQ10* intron. Because some introns that are known to stimulate expression in a position-dependent manner contain few matches to this motif, there must also be other sequences that act similarly.

The ability of the TTNGATYTG motif to boost mRNA accumulation from the 5′-UTR and coding sequences of an intronless construct indicates that splicing cannot be a necessary feature of the mechanism through which it acts. However, the effect of the motif was somewhat higher when within an intron. Thus, while the ability of intron sequences to stimulate gene expression is predominantly splicing independent, splicing may also contribute to an increase in mRNA accumulation. The overall level of expression is likely determined by multiple mechanisms^38,39^.

The observation that the TTNGATYTG motif can stimulate gene expression from coding sequences outside of the context of an intron suggests that other exonic sequences near the 5′ end of genes might be able to stimulate expression by the same mechanism. IMEter scores, which strongly correlate with the ability of an intron to increase mRNA, are generally high in 5′-UTRs and to a lesser degree coding sequences near the start of a gene^40^. The IMEter may thus be a useful tool for identifying potential expression-stimulating sequences in both exons and introns.

Introns with high IMEter scores are often associated with strongly expressed constitutive genes^41^. It is possible that housekeeping genes have evolved sequences throughout their 5′ ends that maximize ubiquitous expression. These sequences may be identified more readily in introns due to the relative ease of generating and studying cDNA. Introns are also under fewer evolutionary constraints than 5′-UTRs and coding sequences and therefore may be more likely to contain regulatory elements. However, the degenerate genetic code does allow for some flexibility. Not all codons are used with the same frequency, and this codon-usage bias can have dramatic effects on gene expression by diverse mechanisms (reviewed in^42^).

N-terminal codon selection is thought to be especially important in determining expression levels. Effects on RNA secondary structure are the largest contributing factor, but still only explain about half of the variation observed^43,44^. In yeast, synonymous mutations at the 5′ ends of genes have been shown to impact nucleosome positioning^45^. Synonymous substitutions also appear to occur less frequently at the 5′ end of genes in mammalian populations (as determined by comparing evolution of the *BRCA-1* gene in humans and dogs)^46^. In addition to codon usage, nucleotide frequency distributions also differ along the lengths of genes, suggesting that promoter proximal sequences may have evolved in response to pressures such as maximizing gene expression^47–49^. Further, optimizing expression by varying codon usage is more effective when adjacent codon pairs, rather than individual codons, are considered^50–52^. It is possible that some of the observed variation in expression associated with codon-usage bias is due to the inadvertent creation or destruction of stimulating sequences like the TTNGATYTG motif in coding regions.

The ability of the TTNGATYTG motif to strongly increase mRNA accumulation from more than 500 nt downstream of the TSS, but not from 1800 nt or more downstream of the TSS or when upstream of the TSS, is difficult to reconcile with known mechanisms of eukaryotic gene expression. The ability of the TTNGATYTG motif to stimulate mRNA accumulation, while small changes to this sequence reduce or eliminate its effect on expression^35^, suggests that there may be a protein such as a transcription factor that binds the motif in a sequence-specific manner. A transcription factor that binds this motif would be unique for genes transcribed by RNA polymerase II in that it only functions when downstream of the transcription start site and activates transcription several hundred nucleotides upstream of its binding site. The TTNGATYTG motif most closely resembles consensus sites for the GATA family of transcription factors^53,54^, but GATA factor-binding sites do not meet the strict positional requirements characteristic of this motif^55–57^.

A second possible way in which the TTNGATYTG motif might boost expression includes effects on local chromatin structure that favor transcript initiation, but this would not explain why this sequence must be downstream of the TSS to stimulate expression or the linear additivity of the motifs in stimulating mRNA accumulation over a wide range.

A third possibility is that the TTNGATYTG motif and other sequences associated with IME influence the transcription machinery during elongation, elevating mRNA production by increasing RNA Polymerase II processivity or the rate of transcription. This mechanism would not account for the ability of the *UBQ10* and *COR15a*11L introns to boost expression in the absence of prior transcriptional activity, either because the gene is normally not expressed in those tissues^58^ or because the usual transcription start sites have been deleted^23^. Furthermore, the stimulatory effects of the stronger *COR15a*11L intron extended over a greater distance than those of the *UBQ10* intron. If the transcription machinery stalls unless it encounters an intron within a certain distance after initiating, it should do so at the same location regardless of the identity of the intron that it failed to reach.

In conclusion, what had been previously characterized as intron-mediated enhancement may not be limited to introns, and it is clearly splicing independent in some cases. The ability of transcribed sequences near the start of genes to affect mRNA accumulation by this mechanism extends beyond introns and may include 5′-UTRs or coding sequences. These transcribed expression-stimulating sequences can be a useful addition to the promoters and enhancers used to regulate gene expression levels in transgenic or synthetic constructs. Introns containing different numbers of the TTNGATYTG motif could be a simple way to vary the expression level of a gene for scientific or practical purposes without altering the structure of the mature mRNA or protein produced.

## Methods

### Cloning of reporter gene fusions

The starting intronless *TRP1:GUS* template for all constructs included a 2.4 Kb *TRP1* promoter fragment that extends from the middle of the upstream gene (At5g17980) through the first 8 amino acids of the third exon of *TRP1* fused to the *E*. *coli uidA* (*GUS*) gene in the binary vector pEND4K^59^. To test the ability of the previously generated *COR15a11L* intron to stimulate expression from four additional locations (Fig. 1), the intron, which is flanked by *PstI* sites^23^, was cloned into previously generated *TRP1*:*GUS* constructs with *PstI* sites either 1095 or 1834 nucleotides downstream of the *TRP1* start codon^22^, or 21 or 324 nucleotides upstream of the *TRP1* start codon^23^.Transgenic plants in which the *COR15a11L* intron is located between the endogenous first and second exons of *TRP1:GUS* were previously described^35^. Other introns flanked by *PstI* sites are efficiently spliced at these locations^22,23,35^.

To introduce the TTAGATCTG motif and TTAGTACTG control motif into exons (Fig. 3), *TRP1* sequences containing the described changes were synthesized by Biomatik (Wilmington, Deleware) and confirmed by sequencing. These fragments were used to replace analogous sequences between a *PstI* site engineered into the *TRP1* 5′ UTR 87nt upstream of the start codon^23^ and a *BamHI* site in the polylinker region connecting the *TRPI* and *GUS* coding sequences.

All fusions were then transformed into *Agrobacterium tumefaciens* C58C1 pMP90 by electroporation and introduced into *Arabidopsis thaliana* ecotype Columbia (Col) by floral dip as described^31^.

### Quantitative comparisons of enzyme activity and mRNA levels

Single-copy transgenic lines were identified, and mRNA levels on RNA gel blots and GUS activity in leaf extracts were measured as previously described^22^. In short, seeds from several dozen lines were screened for a 3:1 segregation ratio (kanamycin resistant: sensitive), and gel blots of DNA digested with restriction enzymes were probed with the *GUS* gene to determine transgene copy number. Single copy, homozygous lines were propagated to the T_3_, T_4_, or T_5_ generation. RNA was extracted from 3-week-old seedlings, grown under constant light in Professional Growing Mix (Sun Gro Horticulture, Agawam, MA) at a density of 500 plants per 170 cm^2^ pot, using the Qiagen RNeasy kit. RNA gel blots were hybridized with a ^32^P-labeled *GUS* probe, and *GUS* mRNA levels in PhosphoImager scans were measured as pixels above background using Image Quant Software as described previously^32^. Quantitative measurements of GUS enzyme activity in leaf extracts were performed as described^22^. The *GUS* mRNA and enzyme activity levels were normalized (to endogenous *TRP1* mRNA levels or total protein, respectively) and compared to the intronless control pAR281^31^. All single-copy lines were used and given equal weight in calculating average expression from a construct.

Statistical differences in gene expression between constructs were analyzed by comparing Log mRNA levels using a mixed model that accounted for blot-to-blot differences, and adjusted for random effects per line and date of mRNA extraction for biological replicates. Residual normality was analyzed using a Wilk Shapiro test and homoscedasticity using a Levene ANOVA. Among the levels of categorical predictors, post hoc comparisons were based on least squares means using a protected least significant difference.

## Supplementary information

Supplementary Material

## Data Availability

All data generated or analysed during this study are included in this published article and its Supplementary Information Files. Sequence data from this article can be found in the Arabidopsis Genome Initiative or EMBL/GenBank data libraries under the following accession numbers: *COR15a* (At2g42540), *TRP1* (At5g17990), and *UBQ10* (At4g05320). All materials and strains are available upon request.
