# Supplementary material for: An intron-derived motif strongly increases gene expression from transcribed sequences through a splicing independent mechanism in Arabidopsis thaliana
Source: Sci Rep. 2019 Sep 24;9:13777. doi: 10.1038/s41598-019-50389-5 (PMC6760150; doi:10.1038/s41598-019-50389-5)

Supplementary Material to accompany:

An intron-derived motif strongly increases gene expression from transcribed sequences through a splicing independent mechanism in *Arabidopsis thaliana*

Jenna E. Gallegos and Alan B. Rose

Supplementary Table S1. Detailed expression data for the COR15a11L intron at different locations.

Supplementary Table S2. Complete cumulative mRNA expression data for published constructs.

Supplementary Table S3. Detailed expression data for the COR15a intron modified to contain different numbers of the TTNGATYTG motif.

Supplementary Table S4. Detailed expression data for the TTNGATYTG motif in the 5'UTR and coding sequences.

Supplementary Figure S1. Complete gel used to make Figure 1b.

Supplementary Figure S2. Complete gel used to make Figure 2b.

Supplementary Figure S3. Complete gel used to make Figure 4b.

Supplementary Table S1. Detailed expression data for the *COR15a* 11L intron at different locations.

Supplementary Table S1a. RNA levels

Intron	Location	Construct	Line	Copy #	RNA gel blots N108	RNA gel blots N109	RNA gel blots N110	RNA gel blots N111	RNA gel blots N1Cheng	Line Avg	Line St dev	Construct Avg	Construct St dev	Count	Biological replicates
COR15a11L	-321	JG25	R3	1		0.49	0.78		0.57	0.61	0.15	0.67	0.14	6	4
			R9	1	0.61	0.67		0.88		0.72	0.14				
COR15a11L	-18	JG36.5	R1 B	1	19.27	27.25	25.96		34.45	26.73	6.22	28.62	5.99	6	4
			R1 D	1		29.21		35.58		32.40	4.50				
COR15a11L	218	NK4	B13	1	16.38	21.39	19.53	19.37	26.2	20.57	3.62	19.53	3.60	7	4
			B16	1		18.71	15.14			16.93	2.52				
COR15a11L	1095	JG79	B10	1		8.87		10.59		9.73	1.22	7.09	3.14	4	4
			R17	1		4.13		4.76		4.45	0.45				
			B2	2	7.2	8.24	8.3	9.81	7.3	8.17	1.05				
			B8	2			10.96	9.83		10.40	0.80				
			B18	2		6.5		9.37		7.94	2.03				
COR15a11L	1834	JG80	B5 C	1	3.86	4.54	3.03		3.76	3.80	0.62	3.76	0.48	6	3
			B5 I	1			3.64	3.72		3.68	0.06				
UBQ10	-321	JG24	B3	1	0.45		0.54		1.14	0.71	0.38	0.71	0.38	3	2
UBQ10	-18	JG36	B9	1	13.8		18.16		21.11	17.69	3.68	17.57	3.01	4	3
			B12	1				17.22		17.22					
UBQ10	218	AR490	G4	1	10.76		16.28		14.56	13.87	2.82	14.08	2.34	4	3
			O1	1				14.7		14.70					
UBQ10	1095	AR448	G1	1	1.52		2.13		0.68	1.44	0.73	1.48	0.60	4	3
			R4	1				1.6		1.60					
UBQ10	1834	AR450	R12	1	1.83		3.01		1.77	2.20	0.70	1.98	0.73	4	3
			R18	1				1.3		1.30					
			Date RNA isolated		11/13/18		1/22/19								

For previously published constructs (all but JG79 and JG80), the values used are only those from current experiments. See Supplementary Table S2 for complete cumulative data.

Supplementary Table S1b. GUS enzyme activity

Intron	Location	Construct	Line	Copy #	GUS enzyme assays		Line Avg	Line St dev	Construct Avg	Construct St dev	Count	Biological replicates
COR15a11L	-321	JG25	R3	1	0.98	1.18	1.08	0.14	0.95	0.24	4	4
			R9	1	0.62	1.03	0.83	0.29				
COR15a11L	-18	JG36.5	R1 B	1	55.34	50.44	52.89	3.46	53.28	2.59	4	4
			R1 D	1	55.61	51.72	53.67	2.75				
COR15a11L	218	NK4	B13	1	35.12	47.46	41.29	8.73	46.82	11.16	4	4
			B16	1	43	61.71	52.36	13.23				
COR15a11L	1095	JG79	B10	1	19.28	30.93	25.11	8.24	17.33	10.17	4	4
			R17	1	8.85	10.27	9.56	1.00				
			B2	2	16.86	18.27	17.57	1.00				
			B8	2		20.57	20.57					
			B18	2	16.25	17.63	16.94	0.98				
COR15a11L	1834	JG80	B5 C	1	5.98	7.36	6.67	0.98	6.60	0.70	3	3
			B5 I	1		6.45	6.45					
UBQ10	-321	JG24	B3	1	0.48	0.45	0.47	0.02	0.47	0.02	2	2
UBQ10	-18	JG36	B9	1	34.48	37.45	35.97	2.10	38.62	4.84	3	3
			B12	1		43.94	43.94					
UBQ10	218	AR490	G4	1	22.91	23.84	23.38	0.66	25.42	3.58	3	3
			O1	1		29.52	29.52					
UBQ10	1095	AR448	G1	1	3.11	3.27	3.19	0.11	3.04	0.27	3	3
			R4	1		2.75	2.75					
UBQ10	1834	AR450	R12	1	5.39	6.9	6.15	1.07	4.86	2.35	3	3
			R18	1		2.29	2.29					
					11/13/18	1/22/19						

Note. JG79 R17 produces roughly half the GUS mRNA and enzyme activity of the known 2-copy lines JG79 B2, JG79 B8, and JG79 B18. JG79 line B10 has similar GUS mRNA levels and enzyme activity as the 2-copy lines, but genomic DNA gel blot analysis with four separate enzymes (*Pst*I, *Bam*HI, *Bgl*II, and *Hin*dIII) indicates a single transgene in both JG79 B10 and JG79 R17. Because the reason for the different GUS expression in lines JG79 B10 and JG79 R17 could not be determined, both were considered single-copy and the values obtained from each were used equally in calculating average construct expression.

Supplementary Table S2. Complete cumulative mRNA expression data for published constructs.

Construct	Intron	Location	Sup. Table	Average	St dev	Count	Reference
JG25	*COR15a* 11L	-321	1a	0.77	0.28	14	23
JG36.5	*COR15a* 11L	-18	1a	26.68	5.9	11	23
NK4	*COR15a* 11L	218	1a, 2a	21.2	5.26	24	35
JG24	*UBQ10*	-321	1a	0.61	0.23	10	23
JG36	*UBQ10*	-18	1a	16.08	3.6	19	23
pAR490	*UBQ10*	218	1a	13.34	2.46	49	32
pAR448	*UBQ10*	1095	1a	1.31	0.39	10	22
pAR450	*UBQ10*	1834	1a	1.66	0.71	9	22
pAR495	*COR15a*	218	2a, 3a	1.7	0.39	45	32
AH3	*COR15a* 6L	218	2a, 3a	13.2	3.25	47	35

Supplementary Table S3. Detailed expression data for the *COR15a* intron modified to contain different numbers of the TTNGATYTG motif.

Supplementary Table S3a. GUS RNA levels

Intron	# of motifs	Construct	Line	RNA gel blot data N103	N104	N105	N106	N107	Line Avg	Line St dev	Construct Avg	Construct St dev	Count	Biological replicates
COR15a	0	AR495	G1	1.87		1.91	1.45		1.74	0.25	1.74	0.25	3	2
			G7	10.62	9.3	11.6		7.5	9.76	1.77				
COR15a 3L	3	KJS3	G11	8.85	8.28	9.68	8.33	7.54	8.54	0.79	9.15	1.44	13	6
			O12		9.97	11.52	7.83	7.88	9.30	1.78				
			B12	12.11			11.89		12.00	0.16				
COR15a 6L	6	AH3	O4				9.06		9.06		12.18	2.71	4	3
			O7	15.67					15.67					
COR15a 8L	8	DKA2	R4	16.21	16.47	17.45	12.6	12.54	15.05	2.31	15.46	2.81	10	4
			Y8	18.06	17.74	19.25	11.9	12.34	15.86	3.46				
COR15a 11L	11	NK4	B13	19.4	19.7	21.35	16.08		19.13	2.21	18.94	1.83	7	4
			B16	18.98		20.12	16.95		18.68	1.61				
			O13	27.61	27.75	28.1	25.33	21.39	26.04	2.82				
COR15a 15L	15	ZJM2	Y3		31.36	34.94	25.29	23.94	28.88	5.17	27.56	3.70	16	8
			Y6		31.73	31.27		26.76	29.92	2.75				
			Y17	26.34	27.86	29.73		21.52	26.36	3.51				
COR15a 20L	20	KJS4	B12	35.15	35.12		32.67	29.97	33.23	2.46	32.83	2.29	9	4
			B19	32.4	32.86	35.17	28.69	33.43	32.51	2.38				
			Date RNA isolated	8/6/18			10/2/18							

For previously published constructs AR495, AH3, and NK4, the values used are only those from current experiments. See Supplementary Table S2 for complete cumulative data.

Supplementary Table S3b. GUS enzyme activity

Intron	# of motifs	Construct	Line	GUS enzyme assays		Line Avg	Construct Avg	Construct St dev
COR15a	0	AR495	G1	1.55	1.81	1.68	1.68	0.18
			G7	17.63	19.36	18.50		
COR15a 3L	3	KJS3	G11	15.95	16.41	16.18	17.91	2.30
			O12	21.85	16.27	19.06		
			B12	23.34	20.99	22.17		
COR15a 6L	6	AH3	O4		19.74	19.74	23.08	3.75
			O7	28.23		28.23		
COR15a 8L	8	DKA2	R4	31.98	26.03	29.01	27.54	3.01
			Y8	25.44	26.69	26.07		
COR15a 11L	11	NK4	B13	31.98	33.78	32.88	36.11	4.47
			B16	42.23	36.44	39.34		
			O13	52.94	52.57	52.76		
COR15a 15L	15	ZJM2	Y3	61.91	55.53	58.72	54.59	4.57
			Y6	55.91	58.54	57.23		
			Y17	52.71	46.58	49.65		
COR15a 20L	20	KJS4	B12	75.45	57.07	66.26	65.56	7.59
			B19	65.9	63.83	64.87		
				8/6/18	10/2/18			

Supplementary Table S4. Detailed expression data for the TTNGATYTG motif in the 5'UTR and coding sequences.

Supplementary Table S4a. GUS RNA levels

Sequence	Location	Construct	Line	RNA gel blot data						line		construct		count	Biological replicates	Statistical group
				JN11	JN12	JN13	JN14	JN15	JN16	Avg	St dev	Avg	St dev			
Cor15a6L	218	AH3	B9		13.04	6.54				9.79	4.60	9.56	4.75	7	4	A
			B12	18.98			7.21	6.53		10.91	7.00					
			O7				7.93	6.72		7.33	0.86					
TTNG AT YTG (6 copies)	5'UTR & CS	JG71	B3	10.63	5.98	5.70				7.44	2.77	7.31	2.29	12	10	AB
			B6	8.71	5.30	5.63				6.55	1.88					
			B9		8.50		4.50			6.50	2.83					
			B10		10.46		6.11			8.29	3.08					
			R6		10.50		5.66			8.08	3.42					
TTNG TA YTG (6 copies)	5'UTR & CS	JG72	B5	0.38	1.02	1.21	0.81			0.86	0.36	0.86	0.30	10	8	D
			B15	0.62	0.77					0.70	0.11					
			R5	0.50	1.02	1.06	1.24			0.96	0.32					
Cor15a intron	218	AR495	G1				1.61	1.44	0.74	55.45	108.37	1.22	0.30	6	4	C
			G9				1.28	1.20	1.06	1.18	0.11					
				8/10/16	11/17/16		12/13/16		1/10/17							

For previously published constructs AH3 and AR495, the values used are only those from current experiments. See Supplementary Table S2 for complete cumulative data.

Supplementary Table S4b. GUS enzyme activity

Sequence	Location	Construct	Line	Gus Enzyme Assays				line		construct	
								Avg	St dev	Avg	St dev
Cor15a6L	218	AH3	B12	15.25		18.63		16.94	2.39	15.05	4.46
			O7		8.71	17.61		13.16	6.29		
TTNG AT YTG (6 copies)	5'UTR & CS	JG71	B3	5.96	4.75			5.36	0.86	8.54	3.59
			B6	10.64	5.5			8.07	3.63		
			B9		5.85	12.55		9.20	4.74		
			B10		6.68	14.76		10.72	5.71		
			R6		6.67	12.07		9.37	3.82		
TTNG TA YTG (6 copies)	5'UTR & CS	JG72	B5	0.73	0.29	0.58		0.53	0.22	0.62	0.21
			B15	0.84	0.45			0.65	0.28		
			R5	0.75	0.45	0.85		0.68	0.21		
Cor15a intron	218	AR495	G1			1.63	1.50	1.57	0.09	1.61	0.13
			G9			1.52	1.79	1.66	0.19		
				8/10/16	11/17/16	12/13/16	1/10/17				

Supplementary Figure S1. Complete gel used to make Figure 1b.

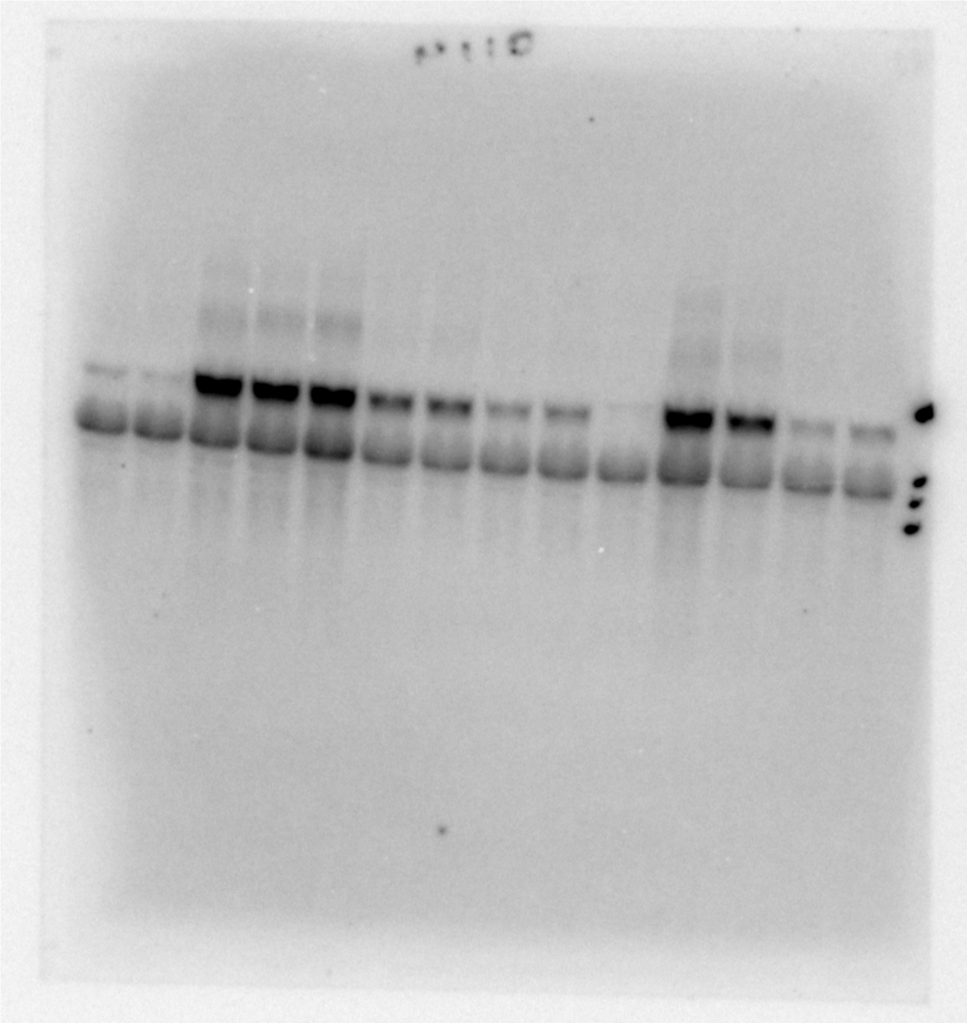

Supplementary Figure S2. Complete gel used to make Figure 2b.

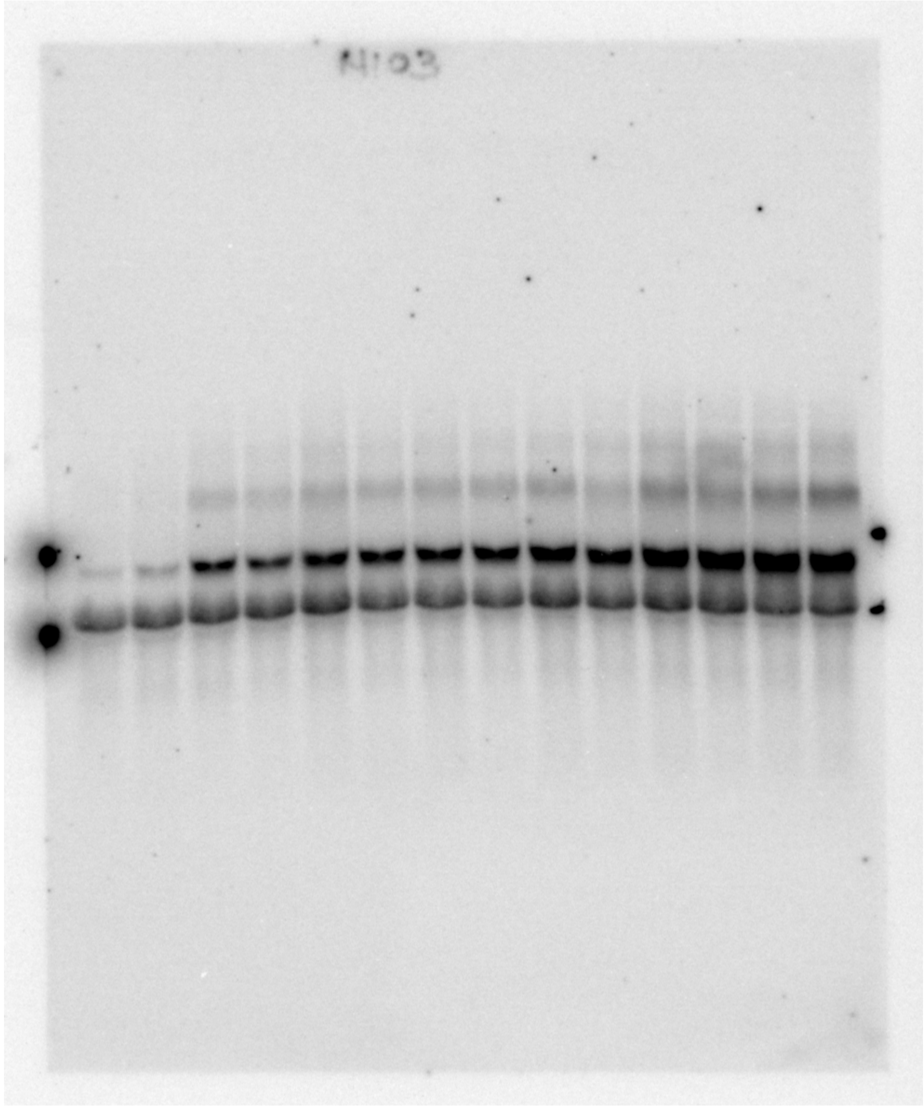

Supplementary Figure S3. Complete gel used to make Figure 4b.

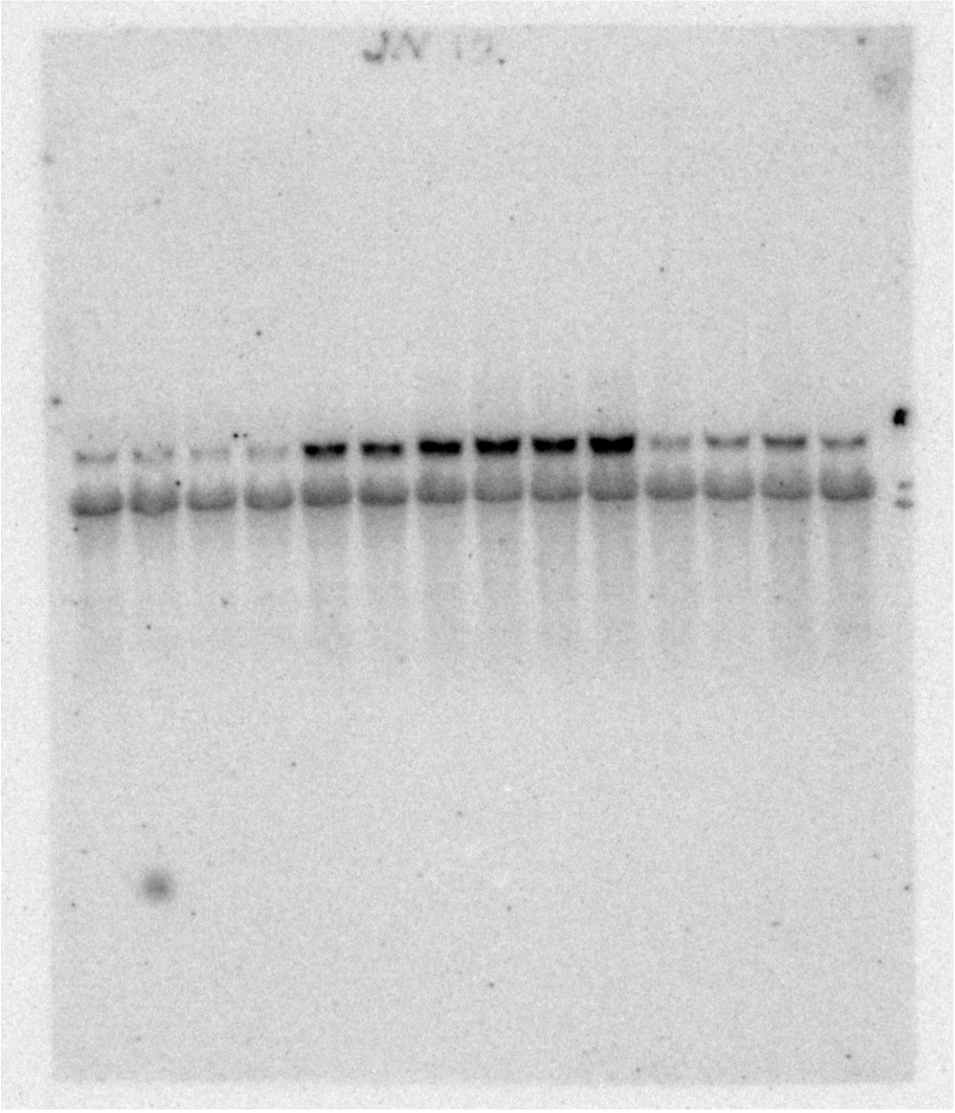

Supplement: Supplementary file 1 — Supplementary Material [file 41598_2019_50389_MOESM1_ESM.pdf]
